# Alpha 1-Antitrypsin Deficiency: A Disorder of Proteostasis-Mediated Protein Folding and Trafficking Pathways

**DOI:** 10.3390/ijms21041493

**Published:** 2020-02-21

**Authors:** Esra Karatas, Marion Bouchecareilh

**Affiliations:** INSERM, CNRS, UMR1053 Bordeaux Research In Translational Oncology, BaRITOn, University Bordeaux, 33076 Bordeaux, France; esra.karatas@u-bordeaux.fr

**Keywords:** Alpha 1-Antitrypsin deficiency, proteostasis, proteostasis network

## Abstract

Human cells express large amounts of different proteins continuously that must fold into well-defined structures that need to remain correctly folded and assemble in order to ensure their cellular and biological functions. The integrity of this protein balance/homeostasis, also named proteostasis, is maintained by the proteostasis network (PN). This integrated biological system, which comprises about 2000 proteins (chaperones, folding enzymes, degradation components), control and coordinate protein synthesis folding and localization, conformational maintenance, and degradation. This network is particularly challenged by mutations such as those found in genetic diseases, because of the inability of an altered peptide sequence to properly engage PN components that trigger misfolding and loss of function. Thus, deletions found in the ΔF508 variant of the Cystic Fibrosis (CF) transmembrane regulator (CFTR) triggering CF or missense mutations found in the Z variant of Alpha 1-Antitrypsin deficiency (AATD), leading to lung and liver diseases, can accelerate misfolding and/or generate aggregates. Conversely to CF variants, for which three correctors are already approved (ivacaftor, lumacaftor/ivacaftor, and most recently tezacaftor/ivacaftor), there are limited therapeutic options for AATD. Therefore, a more detailed understanding of the PN components governing AAT variant biogenesis and their manipulation by pharmacological intervention could delay, or even better, avoid the onset of AATD-related pathologies.

Maintaining the proteome homeostasis or proteostasis through ageing and environmental stresses (heat stress, oxidative stress, exposure to toxic agents) requires the assistance of the proteostasis network (PN) [1,2,3]. This program is assigned to three major arms: protein synthesis and folding, conformational maintenance, and degradation. Consequently, proteostasis program impacts on all biological processes in the different organelles of the cell [4]. Among all these organelles, the endoplasmic reticulum (ER) represents a key “node”, a proteostasis checkpoint for nearly all the secreted proteins [5]. This cellular compartment plays a major role in the synthesis, folding, and structural maturation of more than one-third of all proteins produced in the cell [5]. This ER proteostasis is maintained and ensured by a specific PN consisting of ER-resident enzymes, such as chaperones, glycosylating enzymes, and oxido-reductases. ER PN components monitor the protein-folding status of the ER and initiate measures to maintain ER proteostasis by controlling composition and concentration of the network components. For instance, incompletely and misfolded forms are addressed to degradation by quality control systems, including the ER-associated degradation (ERAD) pathway and autophagy [1,2,3]. Given its central role, the ER proteostasis imbalance is at the root/hallmark of a variety of human diseases. Hypoxia, nutrient deprivation, proteasome dysfunction, or mutations in its client proteins can cause loss in ER proteostasis and lead to accumulation of misfolded proteins and aggregates into the ER, a condition encountered in Alpha 1-Antitrypsin deficiency (AATD) [4].

AATD is a rare genetic disorder with a prevalence of 1 in 2500 that manifests as pulmonary emphysema and liver cirrhosis [6]. AATD is due to mutations in the *SERPINA1* gene (serine proteinase inhibitor, group A, member 1) that leads to the secretion defect of mutant Alpha 1-Antitrypsin (AAT) proteins, which are retained in the ER [7].

Over 100 variants have been identified and are designated by an alphabetic letter according to their isoelectric point [8]. For example, wild-type (WT) AAT protein is referred as to “M” for “medium migration variant” [8]. Among all the AAT mutations identified, the most common and severe disease-causing allele is named Z and occurs in 2% to 3% of the European population. In homozygous patients, the protein is not only inefficiently exported from the ER, but also accumulates as Z-AAT protein aggregates—the main cause of liver disease. The severity of the liver damage ranges from transient neonatal cholestasis to cirrhosis, leading to hepatic transplantation in childhood (mean age 2.5 years old) [9]. Currently, it is impossible to predict which patients with AATD may develop lung or liver issues. For instance, liver biopsy is the only method for assessing the extent of hepatic lesions and liver transplantation is the only therapeutic strategy currently available for AATD-mediated liver disease. Thus, this pathology is the most common genetic cause of pediatric liver disease and the most frequent inherited indication for liver transplantation in the pediatric population [10].

Therefore, a more detailed understanding of the PN components governing AAT-variant biogenesis and manipulating PN components by pharmacological intervention could potentially delay or even avoid the onset of AATD-related pathologies [4,11,12].

The purpose of this review is to provide an overview on ER proteostasis and AATD mediated by the Z variant. We also discuss how manipulation of the ER PN components associated with Z-AAT by pharmacological intervention could be a promising therapeutic strategy.

## 1. ER Proteostasis and Alpha 1-Antitrypsin

### 1.1. Alpha 1-Antitrypsin

AAT is mainly produced and secreted by hepatocytes, but also in a smaller quantity by monocytes, macrophages, pulmonary alveolar cells, and intestinal epithelium.

AAT is a protein encoded by the *SERPINA1* gene (serine proteinase inhibitor, group A, member 1), which is a member of the serpins superfamily. Although originally named because of its ability to inhibit pancreatic trypsin in vitro [13], the principal targets of this serine protease inhibitor are the three neutrophil serine proteases (NSPs): neutrophil elastase, cathepsin G, and proteinase 3 [14]. These proteases act intracellularly within phagolysosomes to digest phagocytized microorganisms [14,15]. Despite their important role in immune response in order to preserve the lung environment against microbial threats, their release following neutrophil accumulation and activation may lead to inflammation and lung matrix destruction [14]. Thus, the production and secretion of AAT is essential in the lung parenchyma to inhibit these NSPs and prevent alveolar tissues from destruction [16].

AAT is the most abundant protease inhibitor in the circulation with around 5 days half-life and a circulation concentration varying between 1.2 to 2 g/L in a healthy individual [16]. This concentration can increase rapidly, up to 2–5 times, during acute phases of inflammation and infection following cytokine (interleukins 1 and 6) and tumor necrosis factor (TNFα) activation [16]. In addition to its major role into inflammation regulation, AAT has been also shown to be involved in angiogenesis and tumor growth processes [17].

As mentioned previously, AAT is encoded by the *SERPINA1* gene, which is located on the long arm of human chromosome 14 (14q32.1) and is composed of six introns (span around 12 kb) and seven exons. These exons are numbered as IA, IB, IC, II, III, IV, and V, where IA and IB contain promoter regions for monocytes and macrophages, whereas IC contains specific promoter for hepatocytes. Consequently, according to the tissue, there is a variability of *SERPINA1* transcripts [18]. Generally, the *SERPINA1* gene is transcribed into pre-messenger RNA, which goes through maturation process (splicing) to generate mature RNA and then localizes to the rough endoplasmic reticulum (RER), the site of translation, folding, and modification.

**Figure 1:**
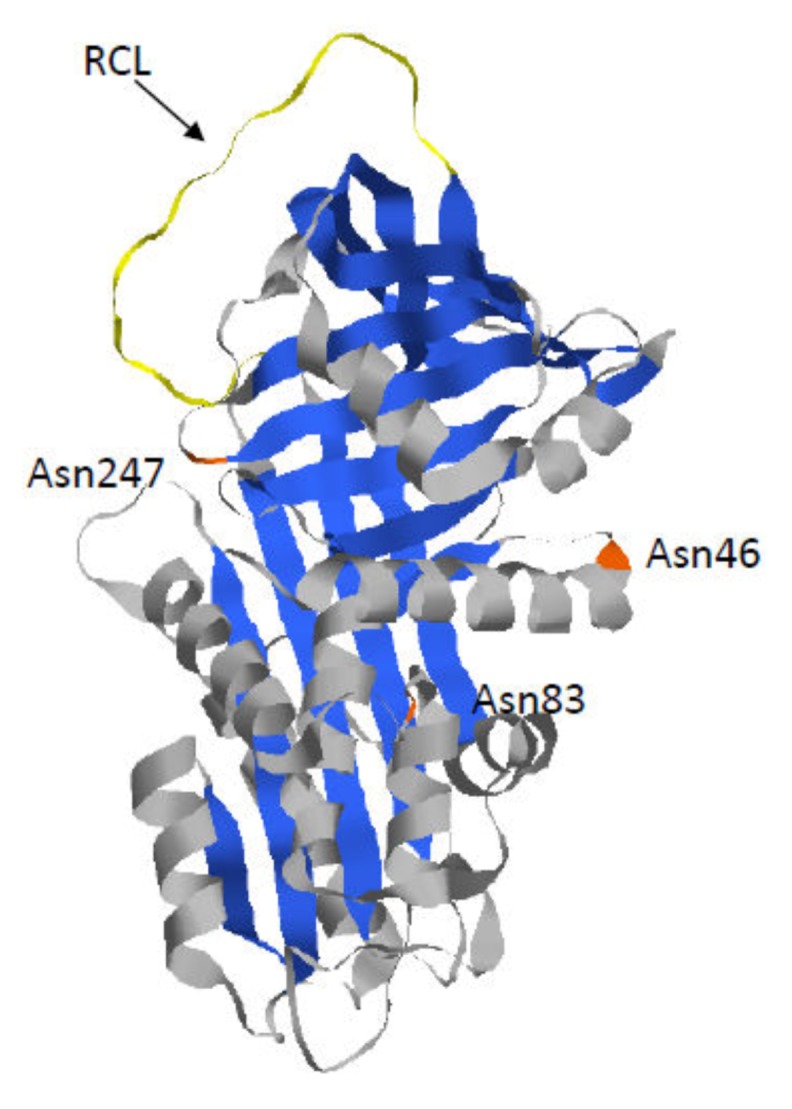
Crystal structure of Alpha 1-Antitrypsin (AAT) (pdb 6I7U). AAT protein is a glycoprotein of 394 residues with three asparagine-linked carbohydrate sidechains at positions 46, 83, and 247 (in orange). The AAT polypeptide chain is composed of three β-sheets (in blue) and nine α-helices (in grey). The reactive center loop (RCL) (in yellow) mediates inhibitory specificity.

The mature AAT is a glycoprotein with three lateral carbohydrate chains. This 52 kDa protein is composed of nine α-helices (A to I), three β-sheets (A to C), and a reactive center loop (RCL), which mediates inhibitory specificity (Figure 1). Indeed, this RCL domain enables formation of a covalent bond between the proteinase targets and AAT. Following this interaction, this bond remains intact while the RCL domain translocates the proteinase and inserts into the middle of the AAT β-sheet A [19,20]. Thus, this conformational change results in a catalytically inactive distorted proteinase.

### 1.2. Z-AAT: The Most Common and Severe Variant

**Figure 2:**
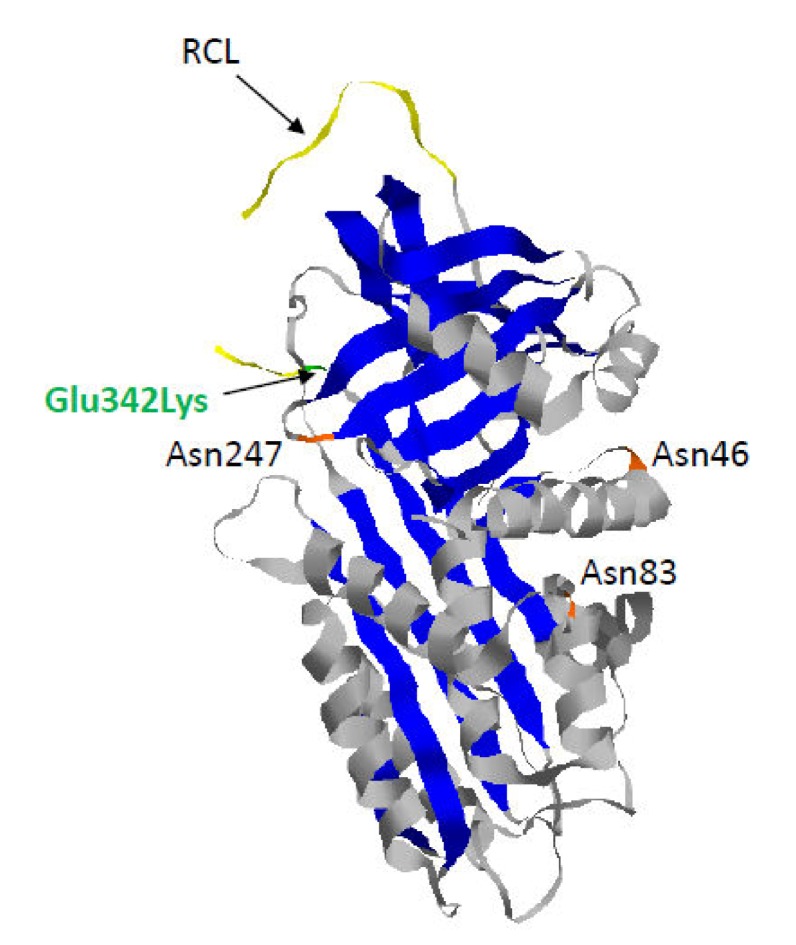
Crystal structure of recombinant human Z-AAT (pdb 5IO1). Z-AAT protein results from the substitution of glutamic acid by lysine at position 342 (Glu342Lys) (in green). As observed for AAT, Z-AAT is composed of three asparagine-linked carbohydrate side-chains at positions 46, 83, and 247 (in orange), three β-sheets (in blue), nine α-helices (in grey), and a reactive center loop (RCL) (in yellow).

The most common and severe variant, named Z, results from the substitution of glutamic acid by lysine at position 342. This mutation is at the head of strand 5 of the β-sheet A and at the base of the RCL that leads to a slow folding of the Z-AAT molecule (Figure 2) [21]. Indeed, during the folding process, this mutation causes an unstable intermediate (M*), which is highly sensitive to temperature changes. It also induces β sheet-A opening, which can accept the RCL of another AAT molecule to form a loop-sheet dimer. The latter can then extend to form longer chains of loop-sheet polymers [22]. This model is based on the “classic” loop-sheet model in which serpin polymers are formed by the intermolecular linkage of the reactive loop of one molecule with the β-sheet A of another [23]. Nevertheless, another serpin polymerization model based on the crystal structure of AAT trimer has been proposed. This model suggests that polymerization, *in vivo*, occurs through a C-terminal domain swap mechanism involving strands 4 and 5 of β-sheet B (s4/5B). Finally, recently, the Z mutation was shown to destabilize the s5A of AAT, leading to an aberrant conformation of the Z monomer. The same defect would disrupt a key step in AAT folding pathway, leading to the pathological Z-polymerization/aggregation via the C-terminal s4/5B domain swap mechanism [24,25].

Overall, even if the conformational process is still debated, it is clear and obvious that the final results of this process ensue in the formation of Z-polymers/aggregates. These structures can accumulate in the ER of hepatocytes and form diastase-resistant, periodic acid-Schiff-positive (PAS + D) inclusion bodies (IB), a hallmark of hepatic injury due to a gain-of-function toxic effect [26,27,28]. This concept of a ‘‘toxic gain-of-function” whereby retention of Z within hepatocytes is responsible for liver disease has been recently supported by the evaluation of the relationship between IB (PAS + D) accumulation and fibrosis stages in vivo; at higher fibrosis stages (individuals with ≥F2), the proportion of biopsies with PAS + D inclusions increases. To sum up, accumulation of IB increases as the stage of fibrosis progresses [29]. Furthermore, Z IBs accumulation may precede chronic portal inflammation and the development of liver fibrosis. In clinical practice, the accumulation of these IBs could be a marker for the population at risk of developing fibrosis [29]. Nevertheless, many questions regarding the formation, composition, and heterogeneity in the distribution and size of IB within hepatocytes still remain to be elucidated (Box 1).

Box 1 **Open-Questions.**

**Inclusion bodies:** Formation, composition and heterogenesity in the distribution and size of Inclusion Bodies.

**Conformational effects of disease Z-AAT mutation:** How does the accumulation of Z-AAT soluble and insoluble forms lead to cellular toxicity? What factors are at the roots or driver of the clinical variability of disease phenotype? Failure to ERGIC-53 binding enhances Z-AAT aggregates

**Z-AAT clearance:** Which type of autophagy (ER-phagy, macroauphagy (mTOR dependent or independent)) is activated by the aggregates? How those types of autophagy work in AATD? What is the mechanismof vesicle or autophagosome formation? Autophagy is a specific response to the accumulation of Z-AAT or rather a secondary process that becomes more important when ERAD and/or the proteasome are overwhelmed?

**PN modulators:** PN modulators strategy could promote the interaction of Z-AAT and ERGIC-53 and consequently increase its secretion? UPR PN modulators can be powerful to avoid AATD disease outcome? Strategy use for CF (lumacaftor, ivacaftor, Dirocafor…) could be successful for AATD?

### 1.3. The Folded AAT vs. the Unfolded Z-AAT in the Secretory Pathway

**Figure 3:**
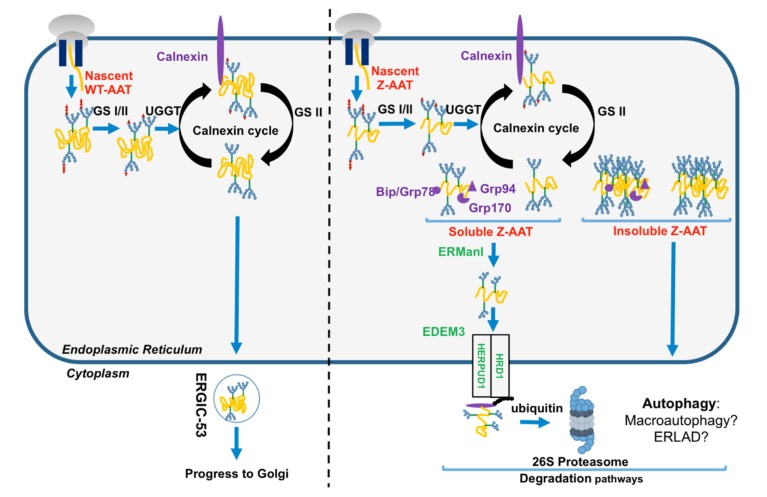
Endoplasmic reticulum (ER) proteostasis network (PN) of the folded AAT and the unfolded Z-AAT protein. Nascent wild-type WT-AAT (left) and Z-AAT (right) are translocated and carried out by quality control system. Compared to the well-folded WT-AAT exported from the ER to the Golgi by endoplasmic reticulum–Golgi intermediate compartment (ERGIC-53) cargo receptor (left), Z-AAT is retained into the ER (right). Z-AAT soluble form is recognized by the ER-associated degradation (ERAD) pathway members (in green), retro-translocated, and degraded by the proteasome. Conversely, the Z-aggregated or insoluble form is degraded by autophagy. AAT: alpha 1-antitrypsin; ERGIC-53: endoplasmic reticulum–Golgi intermediate compartment; ERAD: ER-associated degradation; GRP78: glucose-regulated protein 78; GRP94: glucose-regulated protein 94; GRP170: glucose-regulated protein 170; UGGT: uridine diphosphate (UDP)-glucose:glycoprotein; GS: glucosyltransferase; ERManI: ER α-mannosidase I; EDEM3: ER degradation-enhancing α-mannosidase-like protein 3; HRD1: Hydroxymethylglutaryl-CoA reductase degradation 1 homolog 1/ ERAD-associated E3 ubiquitin-protein ligase; HERPUD1: homocysteine inducible ER protein with ubiquitin-like domain 1.

AAT translation sites are located in exons II, III, IV, and V, where exon II contains start codon (ATG) and exon V contains stop codon (TAA). The translation results in 418 amino acid residues, with a 24-residue peptide signal addressing AAT nascent polypeptide in the RER. This newly translated protein undergoes N-linked glycosylation at positions Asn46, Asn83, and Asn247, and glucose molecules are trimmed from AAT by glucosidase I and II (GS1 and GS2). This event promotes the entrance of the nascent AAT protein into the calnexin (CNX) cycle and its interaction with ER PN proteins such as chaperones CNX, ERp57, and Bip/GRP78 to assure its correct folding and prevent protein aggregation [6,7,12,30]. If AAT folding is correct, the last glucose is removed by GS2 and this protein is exported from the ER by vesicular transport and trafficked to the Golgi apparatus (Figure 3). Luminal protein transport into these vesicles such as AAT, involves a mandatory ER transmembrane protein. The ER protein endoplasmic reticulum–Golgi intermediate compartment (ERGIC-53) has been identified as the main AAT cargo receptor to shuttle AAT from the ER to the Golgi [31]. AAT acquires the last maturation in the Golgi, which modifies the protein, post-translationally, by restructuring glycans (thought to help stabilize the fold) to attain its tertiary structure [17]. AAT appears in globular form before being secreted into the bloodstream.

Conversely to the WT-AAT, which folds rapidly and then interacts only transiently with ER chaperones/PN components, the Z variant slows down the formation of its native monomer, thereby robustly links some ER PN components such as the calnexin-endoplasmic reticulum protein 57 (ERp57)/BiP/glucose-regulated protein 94 (GRP94) [6]. Indeed, misfolded Z-AAT is re-glycosylated by uridine diphosphate (UDP)-glucose:glycoprotein glucosyltransferase 1 (UGGT1), which allows the reinteraction of misfolded Z-AAT with CNX and refolding by ERp57 and BiP [6]. If this misfolded protein cannot attain its mature form, this cycle is stopped following Z-AAT de-mannosylation by the ER α-mannosidase I (ERManI) and ER degradation-enhancing α-mannosidase-like proteins (EDEM), which is the initial step of the ERAD. This pathway designates a cellular pathway targeting misfolded proteins of the ER for ubiquitination and degradation by the proteasome. In this cellular pathway, EDEM3 (ER degradation-enhancing α-mannosidase-like protein 3), degradation enhancer of misfolded glycoproteins in the ER, is implicated in the recognition of ERAD substrates [30,32]. After recognition, ERAD substrates are retro-translocated into the cytosol through a channel formed by some proteins including ubiquitin ligase HMG-CoA reductase degradation 1 homolog (HRD1) and homocysteine inducible ER protein with ubiquitin-like domain 1 (HERPUD1), and are extracted from the ER to the cytosol in order to be transported and degraded by the proteasome (Figure 3) [33]. Finally, in contrast to the WT-AAT, neither Z-AAT nor another Z variant such as the well-known null Hong Kong variant, bind to ERGIC-53, suggesting that ERGIC-53–AAT interaction is conformation-dependent [31]. The failure to the Z variant to bind to ERGIC-53 may contribute to liver and lung diseases associated with AATD by increasing, for instance, Z-AAT aggregation (Box 1).

## 2. Disposal of the Z Mutant

Maintenance of cellular proteostasis requires removal of proteins that fail to attain their native structure. Thus, the protective ER PN is complemented for the preservation of proteostasis by efficient cellular clearance pathways: ubiquitin–proteasome system (UPS) and autophagy [34]. These two pathways functionally cooperate with each other to maintain proteostasis. The Z mutant, as mentioned previously, exists in the ER into two forms: soluble and aggregate. Depending on its status/forms, the Z variant is not supported by the same ER PN components and pathways. Different studies have highlighted the importance of the ERAD pathway in the disposal of the Z-AAT soluble forms. Conversely, the autophagy pathway manages the Z-AAT aggregate forms.

### 2.1. Z-AAT and Proteasome

Proteolysis in eukaryotic cells is mainly maintained and mediated by the ubiquitin (Ub)-proteasome system (UPS). The UPS is a selective proteolytic system in which substrates are recognized and tagged with ubiquitin, a small molecule of 8.6 kDa, for degradation by the proteasome [35]. Briefly, the ERAD pathway targets misfolded proteins to their degradation from the ER to the proteasome. These misfolded proteins are selected by the quality control system, as previously mentioned, and are disposed by the UPS [32]. Due the different compartments of this process (ER, cytosol, proteasome, etc.), ERAD targets (misfolded secretory proteins) require retro-translocation and dislocation trough the ER membrane into the cytosol, followed by proteasomal degradation. ERAD targets are then polyubiquitinated by three enzymes called ubiquitin ligases, which add ubiquitin successively [35]. Polyubiquitinated proteins are recognized by proteasome, which degrades them by removing polyubiquitinated chains via deubiquitinilating enzymes (DUBs enzymes) associated with proteasome.

**Figure 4:**
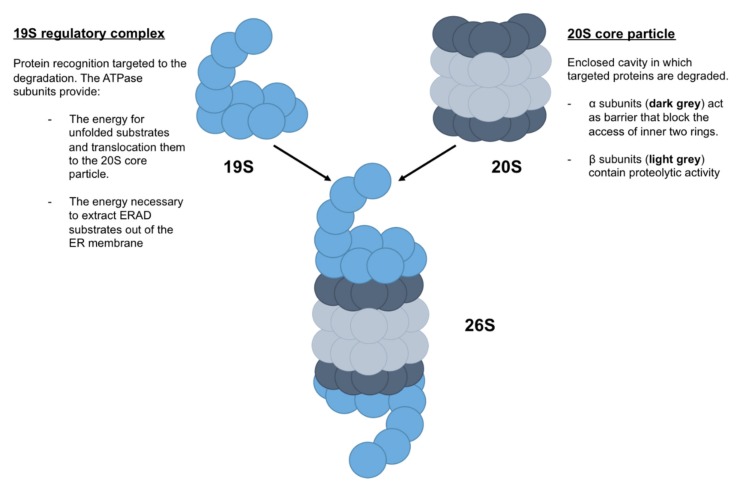
Schematic representation of proteasome 26S. Proteasome 26S, named due to its Svedberg (S) sedimentation coefficient, is formed by a 19S regulatory complex, which recognized proteins targeted to the degradation, and a 20S core particle, which degraded targeted proteins.

The proteasome is a cylindrical complex, composed of (i) the 20S core particle and (ii) the 19S regulatory particle [36] (Figure 4). The 20S core particle is composed of four stacked heptameric ring structures: seven β subunits, which constitute the inner two rings and contain proteolytic activity, and seven α subunits, which constitute the outer two rings and act as a barrier that blocks the access of the inner two rings. Thus, the 20S core particle provides an enclosed cavity in which targeted proteins are degraded. The 19S regulatory particle is composed of a 10-subunit lid and a 9-subunit base, in which six are ATPase subunits that bind directly to the α ring (Figure 4). In addition to the protein recognition targeted to the degradation by 19S regulatory complex, the ATPase subunits provide the energy for unfolded substrates and translocate them to the 20S core particle. Moreover, ATPase subunits may also provide the energy necessary to extract ERAD substrates out of the ER membrane [37].

All the molecular mechanisms leading to Z-AAT retro-translocation from the ER lumen to its degradation by proteasome are not clearly and fully identified. Nevertheless, soluble misfolded Z-AAT is targeted to the cytosolic proteasome through the ERAD pathway involving not only the HRD1–SEL1L complex for its transport across the ER membrane, but also the Sec61 translocon, DERLIN-1 and cytosolic p97/valosin containing protein complexes, Skp1–Cul1–F-box-protein–ubiquitin lipase complexes, and the proteasome [12,32,38,39]. Thus, for instance, silencing the members of the ERAD pathway such as HRD1, HERP, or EDEM3 increase Z-AAT secretion *in cellulo.* HRD1 promotes degradation of Z-AAT and increases its solubility, and conversely HRD1 knockdown enhances Z-AAT accumulation and toxicity [40,41].

Finally, it has also been proposed that proteasome induces the degradation of Z-AAT-soluble monomer through the interaction of Z-AAT and CNX, which induces the polyubiquitination of CNX [42].

### 2.2. Z-AAT and Autophagy

To prevent the accumulation of potentially harmful structures such as the Z-aggregates, cells use another highly conserved degradation pathway, named autophagy, to target large structures/aggregate forms to the degradation by the lysosome [43].

Autophagy, commonly referred to as macroautophagy, requires the fusion between lysosome and autophagosomes (a double-membrane vesicle containing a part of cytoplasm and/or intracellular organelles) [43]. On the basis of this mechanism, it has been inferred that macroautophagy regulates clearance of proteasome-resistant aggregates from the ER, as it does for cytosolic aggregates. This process is defined as ER-phagy and implies the dislocation of luminal aggregates across the ER membrane and/or the capture of ER portions containing them by autophagosomes [44].

Regarding the Z variants and autophagy, it has been shown that administration of autophagic inducers such as rapamycin and carbamazepine (CBZ) in an AATD mouse model increased autophagy and diminished Z-AAT aggregate accumulation [45,46]. The same effect was also observed in another study on the basis of the transcription factor EB master gene (*TFEB*) that regulates the autophagy pathway [47].

Recently, Fregno et al. proposed a new mechanism called ER-to-lysosome-associated degradation (ERLAD). Unlike classic macroautophagy, ERLAD does not require ER capture within autophagosomes but rather relies on vesicular transport, where single-membrane, ER-derived, Z-AAT-containing vesicles release their luminal content within endolysosomes upon membrane–membrane fusion events [48].

All these different studies raise several questions regarding autophagy in AATD, such as which type of autophagy, ER-phagy, or macroautophagy (mTOR-dependent or independent) is activated by the aggregates? How those types of autophagy work in AATD? What is the mechanism of vesicle or autophagosome formation? (Box 1)

### 2.3. Autophagy and Proteasome Crosstalk

The relationship between UPS and autophagy is not fully understood [34,49,50]. It is unclear if autophagy is strictly a parallel degradation system or a compensatory degradation system when the UPS is impaired or overwhelmed [49,51]. This statement is also true concerning AATD. It remains unclear whether autophagy is a specific response to the accumulation of Z-AAT or rather a secondary process that becomes more important when ERAD and/or the proteasome are overwhelmed [52].

Nevertheless, a number of yeast studies reported phenomena that autophagy is activated if ubiquitinated proteins are not properly processed by the proteasome. At low levels/quantity, the Z mutant remains soluble and the ERAD/proteasome pathway disposes of it. Conversely, higher levels of Z-AAT expression induce aggregate formation, thereby activating the autophagy pathway required for their degradation [53].

## 3. Proteostasis Imbalance and AATD-Mediated Liver Toxicity

Z-AAT’s inability to traffic and secrete reflects the loss of folding stability. The latter results in Z-AAT failure to exit the ER and thus to its degradation, contributing to lung disease. In addition, the accumulation of Z-aggregates can also impair the proteasome pathway, triggering induction of autophagy. This likely contributes significantly to liver disease. Indeed, about 10% of the ZZ homozygous patients accumulate Z aggregates that exceed the capacity of the PN to mitigate the problem and the ensuing intracellular injury cascade, cell death, and chronic liver damage [52]. Because they all share the same ZZ genotype, Z-AAT retention and/or accumulation are not sufficient to cause liver damage because not all ZZ patients develop hepatic injuries. Moreover, the main classic environmental factors such as alcohol associated with liver injury cannot be advanced during childhood. Thus, the presence of modifier genes in the onset of severe pediatric AATD liver disease is now well recognized but still remains to be identified [9,33,54]. Among all the potential candidates, several publications suggest that genetic factors affecting the efficiency of the disposal pathways (UPS and/or autophagy) might act as potential modifiers of AATD liver disease outcome. In agreement with this hypothesis, it has been shown that Z-AAT degradation is significantly slower in cells from AATD patients with liver disease than in cells from AATD patients without liver disease, suggesting that disposal pathways seem relatively inefficient in ZZ individuals presenting with a liver disease phenotype [55,56,57]. Haddock et al. have shown in an AATD mouse model that, compared to WT, ZZ mouse liver extracts had about fourfold more polyubiquitin conjugates with no apparent change in the levels of the 26S and 20S proteasomes [52]. This suggests that Z-AAT aggregates indirectly impair degradation of polyubiquitinated proteins at the 26S proteasome level, compromising substrate delivery to the proteolytic core [52]. In addition, two genetic studies, a candidate gene-sequencing strategy and a genome-wide association study have pinpointed some potential genetic modifiers in the ERAD pathway as potential candidates in AATD liver disease [33,54]. Difference in ERmanI expression is associated with an earlier age-of-onset for end-stage liver disease, and *HERPUD1 R50H* and *HFE H63D* variants are associated with the advanced liver disease component of AATD [33,54].

Interestingly, the unfolded protein response (UPR), a part of the ER PN, is activated following accumulation of unfolded proteins within the ER [58]. The goal of this response is to (i) increase the disposal of misfolded proteins; (ii) reduce the amount of nascent proteins in the ER by inhibiting the translation; and finally, (iii) improve protein folding by increasing, for instance, chaperone transcription [58]. Interestingly, accumulation of the Z variant alone into ER hepatocytes fails to activate this adaptive response [59]. It has been proposed that Z-AAT aggregates maintain their regular structure and, therefore, are not recognized as misfolded structure by chaperones and do not induce alone the activation of the UPR. Nevertheless, the accumulation of Z-AAT aggregates renders the cells hypersensitive to ER stress [59]. This is well illustrated in Joly et al. study [33]. In this work, the authors have demonstrated that impairment of the ERAD machinery induces an overload of Z-AAT soluble and insoluble forms into the ER, whereas UPR-mediated cell death was hyperstimulated. Even if the UPR is not induced by the Z-AAT aggregates alone, this adaptive pathway represents a major player in the liver disease pathogenesis upon a second event/hit, observed in 10% of AATD patients [33].

To date, no autophagic factors have been identified that are be involved in AATD liver disease. Nevertheless, the administration of the two autophagic enhancers, CBZ and rapamycin, have been associated with reduction of the hepatic Z-AAT load, inflammation, and hepatic injury, suggesting that autophagy may represent another potential mediator of AATD liver disease [45,46].

In summary, many other factors are likely to be able to modify AATD-associated liver disease and have to be elucidated. Further genome-wide association studies may allow for exploration and resolution of the role that these pathways play, and more importantly, identify these genetic modifiers in future.

## 4. Proteostasis Modulators in Correction of AATD

An adjustment of the PN environment through proteostasis regulators to promote folding, avoid ERAD degradation, and enhance autophagy raises the possibility that multiple opportunities exist to reduce or even block the phenotypes associated with AATD diseases [12,17].

This hypothesis has been tested and validated for another disease with misfolded proteins —Cystic Fibrosis (CF). This pathology is an autosomal recessive disease, affecting approximately one in 2500 live births caused by mutations in a gene encoding a transmembrane protein, the cystic fibrosis transmembrane conductance regulator (CFTR) [60]. Similar to AAT protein, to acquire its fully folded native structure, nascent CFTR is first carried out by the ER quality control system that allows the exit of folded proteins whilst targeting misfolded CFTR to degradation [61]. At the Golgi level, CFTR is also modified and reaches the post-Golgi compartments to be delivered to the plasma membrane where it functions as a chloride/bicarbonate channel across exocrine gland epithelial cell membranes. Thus, this protein affects secretory function in multiple organs (lungs, liver, pancreas, etc.), and its function is determined by its ability of membrane channel opening and its appropriate conductance of anion movement across this channel [60].

Over 1500 *CFTR* gene mutations have been identified, resulting in defective CFTR proteins (defective anion channel function, amount of CFTR protein present at the cell surface, etc.). Among all the organs that express CFTR, the lungs are the most severely affected by CFTR mutations, leading to death in 90% of patients. Indeed, defective CFTR results in mucus obstructing airways, leading to serious lung infections and huge neutrophil infiltration and inflammation contributing to tissue destruction [62]. Thus, treatments for CF have expanded massively over recent decades, in particular with the identification of specific PN modulators. These modulators are the only treatments approved for CF patients and represent the most important advance in any respiratory disease. For instance, the G551D-CFTR mutation impairs the gating activity of the protein at the cell surface. In such cases, this variant is addressed to the cell surface but the gates do not open correctly in order to permit ion exchange. PN potentiators such as Kalydeco (an Food and Drug Administration (FDA)-approved drug developed by Vertex Pharmaceuticals) or PTI-808 (developed by Proteostasis Therapeutics) have been identified as mitigating this defect [63].

The most common CFTR mutation worldwide, with approximately 45.3% of CF patients in the United States—a three base pair deletion, ΔF508—results in abnormal folding, leading to its degradation by the proteasome and a lack of CFTR proteins to the apical cell membrane. Conversely to the G551D-CFTR mutation, ΔF508 needs a combination of two modulators (lumacaftor and ivacaftor, also referred to as Orkambi) to correct both protein trafficking and channel gating abnormalities [60].

All these important findings and advances in CF treatments could be applied to develop treatment for AATD. Chemical chaperones such as trimethylamine N-oxide, glycerol, kifunensin (mannosidase I and II inhibitor), or castanospermine (glucosidase inhibitor) have already been identified for their ability to stabilize the folding and/or rescue the traffic of Z-AAT [30,64,65,66]. For instance, the inhibitors kifunensin and castanospermine alter the interaction between Z-AAT and CNX and avoid the targeting of this variant to ERAD/UPS pathways, leading to an increase in Z-AAT traffic and secretion. Nevertheless, the most promising result has been with histone deacetylase inhibitors such as sodium 4-phenylbutyrate (4-PBA) or suberoylanilide hydroxamic acid (SAHA) [30,66]. These two inhibitors are effective in increasing the secretion of functionally active Z-AAT *in cellulo.* Only 4-PBA has been assessed in a clinical trial in individuals with Z-AATD, but was shown to be ineffective [67]. SAHA effects have been evaluated/monitored only in cell lines that support further evaluation in animal models of disease and/or in humans [30].

All the drugs referred here are able to improve the folding of Z-AAT, but another hallmark of the disease is the aggregation. Some PN modulators have also been identified as reducing or blocking the formation of these structures [68,69]. Some of these modulators are directly based on AAT conformation. As previously explained, the formation of AAT aggregates implicated a hydrophobic pocket, which is delimitated by strand 2 of β-sheet A and helices D and E. This hydrophobic pocket accepts an exogenous reactive loop peptide leading to AAT aggregation. Consequently, this conformation has been used to prevent aggregation. For example, a mutation on strand 2 of β-sheet A induces a reduction in the aggregate formation and rescue of Z-AAT secretion [70]. In addition, another strategy based on virtual ligand screening has allowed the discovery of small peptides that target this hydrophobic pocket and the reactive central loop to prevent Z-AAT aggregation [71]. Recently, a new approach based on monoclonal antibodies (mAb4B12) was shown to block Z-AAT polymer formation without compromising its inhibitory activity [72]. Despite promising results, all these strategies need further experiments in cell and animal models.

Finally, PN modulators that are able to regulate the degradation pathway, such as those that stimulate autophagy, could also represent a key strategy to prevent AATD liver disease. In 2010, Hidvegi et al. demonstrated that the FDA-approved drug and autophagic inducer named CBZ enhanced autophagic (independently of mTOR) and proteasomal degradation of Z-AAT, leading to reduction of the intrahepatic PAS + D inclusions, liver fibrosis, and inflammation in an AATD mouse model [72]. In humans, CBZ has been assessed in a randomized, controlled clinical trial in individuals with severe liver disease (NCT01379469). This clinical trial will be finished this coming year. Following this precept paper supporting the development of autophagy enhancers to treat AATD liver disease, other autophagy enhancers have been identified on the basis of different animal models (*C. elegans*, mouse), but all these drugs need further validation of their efficacy in humans [47,73,74,75,76].

## 5. Conclusions

Current therapeutic management for AATD patients with lung issues involves augmentation therapy. Nevertheless, this strategy of treatment is expensive and the cost–benefit ratio is still debated. Currently, there is no specific treatment of AATD liver disease other than standard liver supportive care and liver transplantation in severe cases.

Before genome editing becomes safe and is approved as a clinical tool in genetic diseases, the identification and administration of PN modulators (as in CF patients) represents a promising therapeutic strategy. However, a full understanding of the complete proteostasis network of Z-AAT is mandatory. The establishment of this PN may lead to the identification of signaling pathways and molecules that may be manipulated to increase the folding of Z-AAT soluble forms, facilitate secretion, increase Z-AAT aggregate forms, and reduce liver fibrosis. Given the different Z-AAT forms (soluble and aggregate) and pathways involved in the management of these forms, a combination of drugs—as in CF treatments—could be more successful. For instance, it would be interesting to test the synergistic properties of autophagic drugs dependent and independent of the mTOR pathway such as CBZ and rapamycin. A “triple combination” therapy targeting folding, degradation, and prevention of aggregation could be an eagerly awaited successful treatment for AATD.

## Abbreviations

AAT	Alpha 1-antitrypsin
AATD	Alpha 1-antitrypsin deficiency
PN	Proteostasis network
ER	Endoplasmic reticulum
ERAD	ER-associated degradation
NSPs	Neutrophil serine proteases
RCL	Reactive center loop
PAS + D	Periodic acid–Schiff-positive diastase
GS1 and GS2	Glucosidase I and II
CNX	Calnexin
ERGIC53	Endoplasmic reticulum–Golgi intermediate compartment
ERp57	Calnexin–endoplasmic reticulum protein 57
GRP94	Glucose-regulated protein 94
UGGT1	Uridine diphosphate (UDP)-glucose:glycoprotein glucosyltransferase 1
ERManI	ER α-mannosidase I
EDEM	ER degradation-enhancing α-mannosidase-like proteins
HRD1	HMG-CoA reductase degradation 1 homolog
HERPUD1	Homocysteine inducible ER protein with ubiquitin-like domain 1
UPS	Ubiquitin–proteasome system
DUBs enzyme	Deubiquitinilating enzyme
CBZ	Carbamazepine
TFEB	Transcription factor EB master gene
ERLAD	ER-to-lysosome-associated degradation
UPR	Unfolded protein response
CF	Cystic fibrosis
CFTR	Cystic fibrosis transmembrane conductance regulator
4-PBA	Sodium 4-phenylbutyrate
SAHA	Suberoylanilide hydroxamic acid
